# Coinfection of *Leishmania (Viannia) braziliensis* and *Streptococcus pneumoniae* in Multiple Cutaneous Lesions

**DOI:** 10.1371/journal.pntd.0004388

**Published:** 2016-03-10

**Authors:** Paulo R. Cortes, Laura S. Chiapello, David Dib, Monica V. Herrero, Carmen T. Nuncira, Carlos De Petris, Jose Echenique

**Affiliations:** 1 Departamento de Bioquímica Clínica (CIBICI-CONICET), Facultad de Ciencias Químicas, Universidad Nacional de Córdoba, Córdoba, Argentina; 2 Hospital Pediátrico del Niño Jesús, Córdoba, Argentina; 3 Hospital Córdoba, Córdoba, Argentina; Institut Pasteur, FRANCE

## Case Presentation

A 12-year-old girl was seen by a physician in Frias (Santiago del Estero Province, northern Argentina region) due to a lesion in her left leg that presented a painless papule, which later became ulcerative. The mother referred that the first lesion appeared after an insect bite in the left leg of her daughter, but she did not remember any febrile episode. She reported no other symptoms or past medical history associated with skin ulcerations, diabetes, recurrent ear infections, or chronic diseases. In a few days, new lesions with similar characteristics appeared on her arms and legs. The first clinical impressions were ulcers caused by group A beta-hemolytic streptococci or staphylococci. No bacteriological cultures or other microbiological studies were performed to determine the putative cause of those lesions. Nevertheless, she received an empirical antibiotic treatment, such as topical rifampicin (3 applications/day of a 100 mg/ml solution) concomitantly with penicillin G benzathine (1,200,000 UI, one dose, i.m.) cephalexin (500 mg orally every 12 h for 14 days) and ciprofloxacin (500 mg orally every 12 h for 7 days), which were administered sequentially due to clinical worsening of the skin lesions. After 2 weeks, the physicians could not observe any lesion improvement after these treatments, and judged by the evolution of lesions, they suspected a *Leishmania* infection and interrupted antibiotic treatment. The patient was transferred to the Hospital Pediatrico del Niño Jesus (children’s hospital, Cordoba, Argentina) for an accurate diagnosis.

## Diagnosis

**Fig 1:**
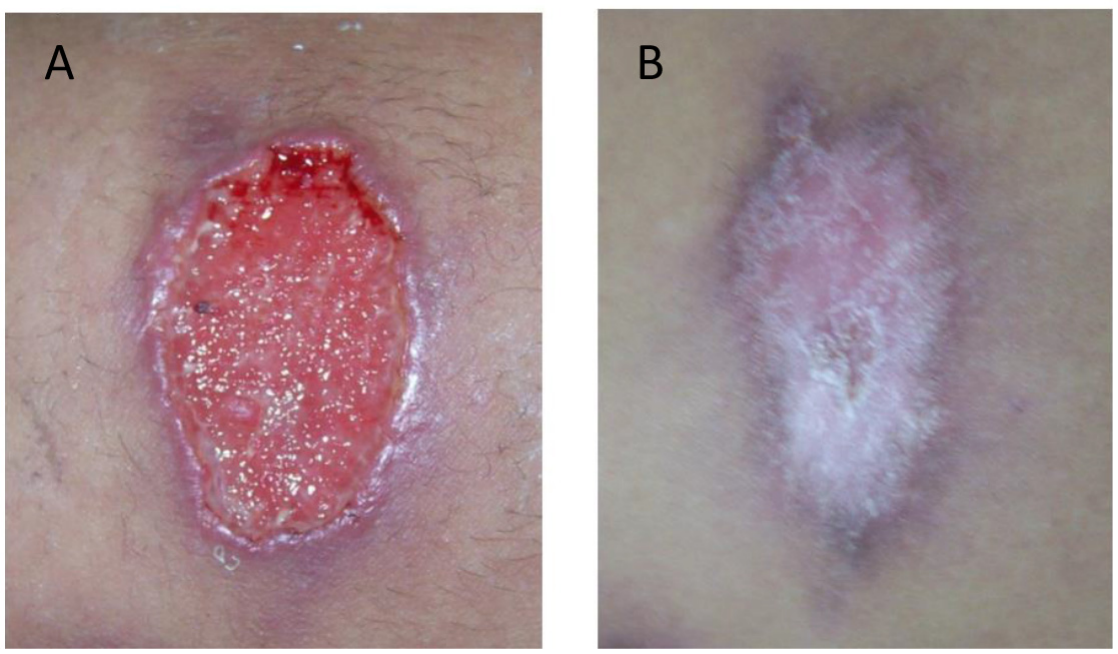
Skin lesion caused by *Leishmania (Viannia) braziliensis* and coinfected with *Streptococcus pneumoniae*. (A) Image of an untreated ulcer when the patient was admitted at the hospital. (B) Image of the same ulcer shown in panel A after the treatment with antibiotics (rifampicin/trimethoprim) and meglumine antimoniate (Glucantime).

On admission, the patient presented multiple skin ulcers on the left leg and in both arms, and she was hospitalized several days for diagnosis and treatment. The clinical examination showed oval ulcers with an average diameter of 3 x 5 cm, hard edges, erythematous halos, painful to the palpation, granular aspect, and covered with a yellowish secretion (Fig 1A).

These ulcers were located on the inner face of the left leg (*n* = 2), the inner face of the right thigh (*n* = 1), and the inner face of the right arm (*n* = 3). Histopathological analysis of lesion biopsies noted abundant fibrin deposition, neutrophil granulocytes, erythrocytes, and necrotic tissues. Neoformed blood vessels, endothelial cell proliferation, and young connective tissues were also observed in subjacent samples. Deep and peripheral samples of lesions showed inflammatory infiltrates constituted by lymphocytes and plasma cells. All these findings were compatible with a nonspecific chronic inflammation. Initial laboratory studies revealed a normal number of white blood cell count (8,700/μl with 69% granulocytes and 26% lymphocytes) and increased erythrocyte sedimentation rate (ESR [40 mm/h]), hemoglobin 139 g/liter, hematocrit at 42 liter/liter and normal blood glucose (95 mg/dL) and urea (20 mg/dL) levels. Once biological samples from lesions were obtained for microbiological tests, the patient received an empirical treatment with rifampicin/trimethoprim (300/80 mg orally every 12 h for 15 days), topical treatments of skin lesions with fusidic acid cream (2%), and povidone/iodine solutions every 12 h.

**Fig 2:**
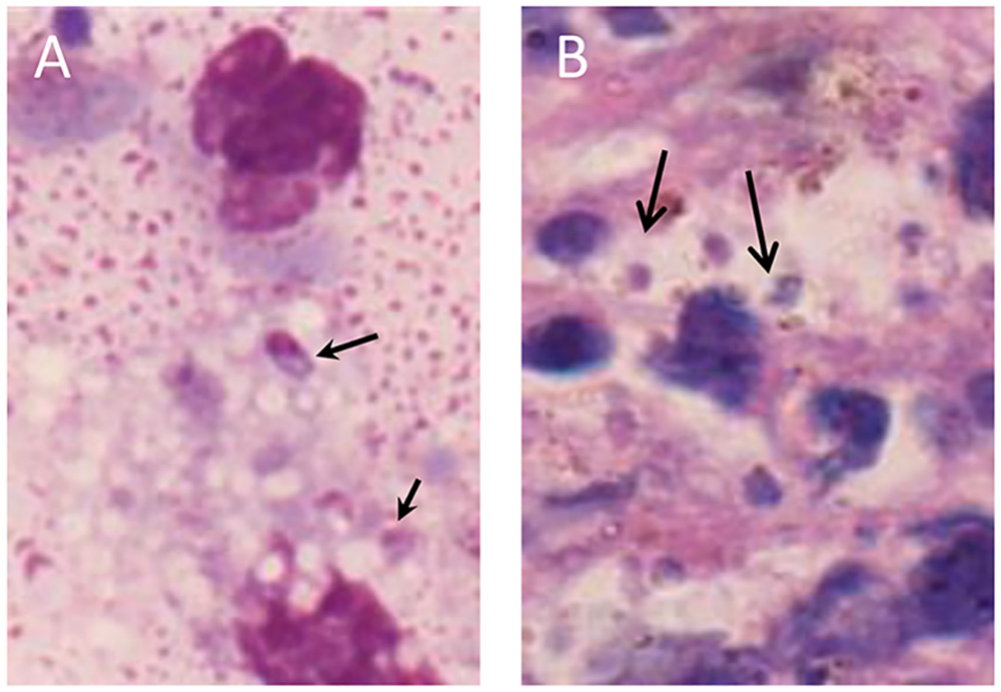
Microscopic identification of *Leishmania* amastigote forms. Biological samples obtained from ulcer were stained by Giemsa technique. (A) image of a peripheral lesion biopsy; (B) image of a deep lesion biopsy. The amastigote forms are indicating by arrows.

To determine a putative *Leishmania* infection, Giemsa-stained thin smears of dermal scrapings were analyzed, which revealed amastigotes inside macrophages, consistent with leishmaniasis (Fig 2).

Furthermore, sterile biopsy specimens for culture were remitted to the Instituto Nacional de Parasitología “Fatala-Chaben.” Immediately, an antiprotozoal treatment with meglumine antimoniate (Glucantime, 20 mg/kg/day i.m. and 1 mg/kg/day intralesion for 1 month) was administered. Then, *Leishmania* promastigotes were identified after 10 days of culture [1], and the amastigotes isolated from lesions were identified as *Leishmania (Viannia) braziliensis* by molecular tests, which is one of the most common *Leishmania* species circulating in Santiago del Estero, Argentina [2].

**Fig 3:**
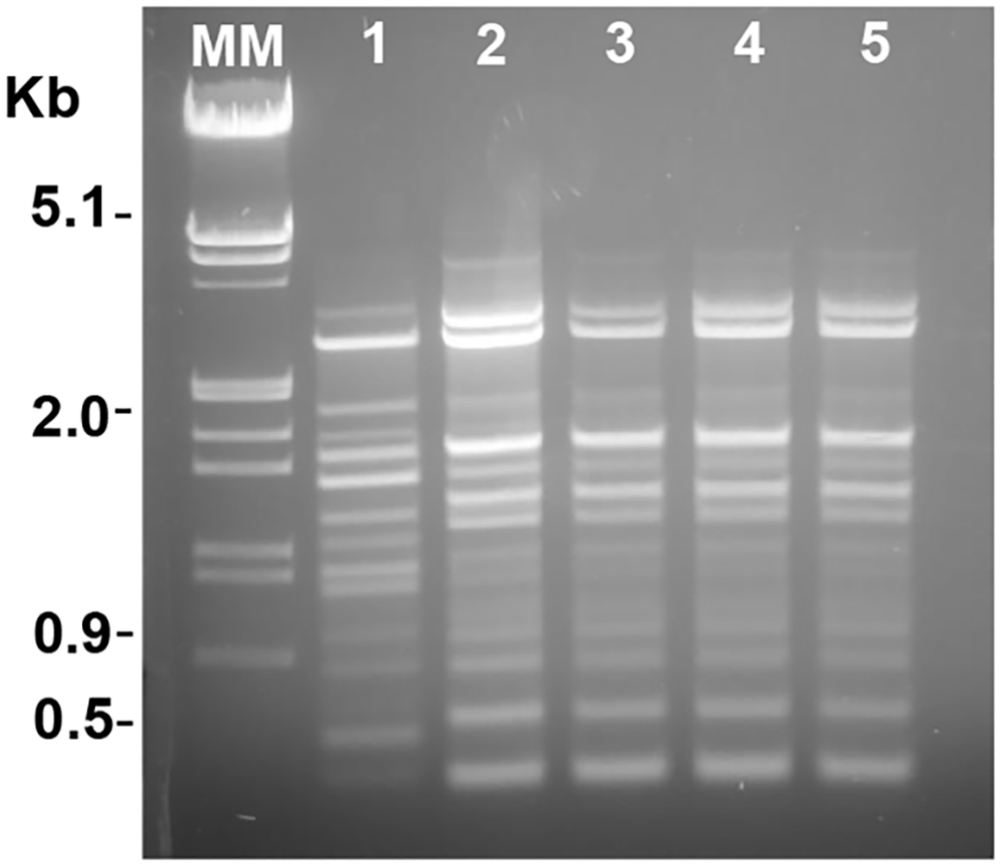
BOX-PCR DNA profiles of *S*. *pneumoniae* isolates. Four pneumococcal strains (lines 2–5) isolated from different ulcers showing genetic identity between them and the same clonal origin. DNA from R6 pneumococcal strain was used as control (line 1). MM, molecular DNA marker (phage λ DNA digested with HindIII/EcoRI).

To detect a possible bacterial coinfection, blood cultures were taken before initiation of empirical antibiotic treatment, but the results were negative. However, the purulent material obtained from lesions was cultured in blood agar plates, and alpha-hemolytic colonies grew, showing a clear mucoid phenotype. Unexpectedly, bacterial strains recovered from four lesions were identified as *Streptococcus pneumoniae* by classical tests, such as optochin susceptibility and bile solubility. This was confirmed by PCR amplification of specific pneumococcal genes, such as *lytA*, *psaA*, *ply*, and *sodA*, as described [3,4]. In addition, the *sodA* gene was partially amplified and sequenced (Macrogen Inc. Seoul, Korea) [3]. The DNA sequences were analyzed in the GenBank database and they showed 99.99% of homology with the *S*. *pneumoniae sodA* gene, confirming the identification of this pathogen. The pneumococcal strains were also serotyped by the Quellung reaction [5] at the Instituto Nacional de Enfermedades Infecciosas (“Carlos Malbran”), and they were identified as serotype 3. To determine the putative origin of these isolates, they were also analyzed by BOX-PCR using a unique BOXA1R primer and following the protocol described [3]. BOX-PCR is a molecular technique that amplifies, by PCR, a DNA-repetitive element named BOX, which is used for epidemiological studies of *S*. *pneumoniae* The BOX patterns showed identical profile (Fig 3), suggesting a clonal relationship between the isolated strains.

The antibiotic susceptibility tests for the pneumococcal strains were carried out using agar diffusion methodology and E-tests for β-lactams antibiotics following the guidelines of the Clinical and Laboratory Standards Institute [6]. The isolates were susceptible to penicillin (Minimal Inhibitory Concentration [MIC]: 0.006 mg/L) and cefotaxime (MIC:<0.006 mg/L) determined by E-test, and to erythromycin, rifampicin, trimethoprim-sulphamethoxazole, clindamycin, chloramphenicol, ofloxacin, and only resistant to tetracycline as determined by disk diffusion, indicating that the empirical antibiotic treatment administered on admission (rifampicin/trimethoprim) was appropriate. After that, the patient was discharged. A month later, when she came back to the hospital for a follow-up appointment, the ulcer lesions were completely healed (Fig 1B), and she did not present any side effects from the medication. In this manuscript, the patient has given written informed consent for publication of her case details.

## Case Discussion

Leishmaniasis is an infectious disease caused by a protozoan microorganism transmitted to humans via the bite of a female sand fly. It is disseminated in five continents and causes more than 30,000 deaths every year. The World Health Organization (WHO) estimates that approximately 350 million people are at the risk of contracting this disease [2]. Different species of *Leishmania* are responsible for cutaneous and visceral diseases that are endemic to tropical and subtropical regions in 98 countries. Cutaneous leishmaniasis is the most common disease that is caused by all *Leishmania* species that are pathogenic to humans and is considered endemic in northern Argentina [1]. WHO also estimates that 0.7–1.2 million new cases of cutaneous leishmaniasis occur annually [7].

It is known that *L*. *(V*.*) braziliensis* normally express virulence factors to disrupt the natural skin barrier to establish cutaneous leishmaniasis [8], and these lesions predispose to coinfections of bacterial pathogens (Table 1) [9–17].

**Table 1:** Bacterial species that coinfect skin ulcers caused by cutaneous leishmaniasis.

Bacterial species	References
*Bacillus* spp.	[16]
*Bacteroides fragilis*	[15]
*Enterobacter* spp.	[14,15,16,17]
*Enterococcus* spp.	[9,10,14]
*Escherichia coli*	[9,11,15,16]
*Klebsiella* spp.	[9,11,14,15,16,17]
*Micrococcus* spp.	[14]
*Morganella morganii*	[14]
*Mycobacterium ulcerans*	[13]
*Peptostreptococcus* spp.	[15]
*Prevotella* spp.	[15]
*Proteus* spp.	[9,11,15,16,17]
*Pseudomonas aeruginosa*	[9,12,14,15,16]
*Staphylococcus* spp.	[11,14,16]
*Staph*. *aureus*	[9,10,11,14,16,17]
*S*. *agalactiae*	[15]
*S*. *pneumoniae*	This work
*S*. *pyogenes*	[9,14,15,16]

Here, we report the first case of coinfection with *Leishmania and S*. *pneumoniae*. This bacterial pathogen resides normally in the human upper respiratory tract, but it is also the causal agent of infections in children and adults such as otitis, sinusitis, pneumonia, septicemia, meningitis, and it is also isolated from skin [18]. Pneumococcal skin infections are more frequently found in immunocompetent patients associated with HIV, lupus erythematosus, diabetes, burns, cancer, and alcoholism. Listed by order of relevance, the clinical sources of these infections were surgical wounds, burns, cellulitis, pyomyositis, fasciitis, and abscess infections [18]. Based on these clinical features, we cannot discard that *S*. *pneumoniae* contributed to the infectious process of lesions caused by *Leishmania*.

Regarding the origin of bacterial infection, the pneumococcal strains isolated from different *Leishmania* skin lesions appear to have the same source of contagion. We think that the pneumococcal isolates that infected skin ulcers came from the patient`s nasopharynx, but we don’t have evidence to discern whether these strains were part of the patient’s microbiota or were acquired in the community. We cannot rule out the possibility that the patient was a nasal carrier of the pneumococcal isolates found in the skin lesions, and this fact is related to pneumococcal survival after the empirical antibiotic therapy. The patient was initially treated with cephalexin and ciprofloxacin, because the lesions appeared to be caused by *S*. *pyogenes* or *Staph*. spp. However, these antibiotics are not indicated for pneumococcal infections because they are less active against this pathogen. She was also treated with penicillin G that is effective for the treatment of pneumococcal infections. Nevertheless, it was reported that penicillin concentrations in saliva were much lower than the corresponding serum concentrations during penicillin therapy, indicating that decolonization is not possible with this antibiotic, and that *S*. *pneumoniae* is able to survive in nasopharynx under these conditions [19]. For these reasons, we think that the patient possibly was a nasal carrier after the antibiotic treatments. These bacterial isolates were identified as serotype 3, which was underrepresented in pneumococcal carriage studies [20], but it was one of the most frequent serotypes isolated from pneumococcal invasive disease in Argentina [21]. There is also the possibility that the patient had acquired pneumococcal strains in the community when antibiotics were interrupted.

Concerning the way by which *S*. *pneumoniae* coinfected these ulcerative lesions caused originally by *Leishmania* on the arms and legs by the nasopharynx, we propose that this bacterial pathogen was disseminated through contaminated respiratory tract secretions like saliva when the patient exhaled or coughed onto skin lesions. Once the first skin lesion was coinfected with *S*. *pneumoniae*, the other ulcerative lesions could have been coinfected by scratching. On the other hand, although the patient did not report clinical symptoms compatible with septicemia or pneumonia before or after her hospitalization, there is a possibility that this strain had been disseminated by a hematogenous spread from the first coinfected lesion. In this sense, Kalima et al. [22] reported that 30/34 cases of pneumococcal infections, where skin was the primary site of infection, had bacteraemia. Thirty percent of these cases did not present underlying predisposing conditions. In addition, these coinfected lesions also represented a risk factor for the invasive disease development caused by *S*. *pneumoniae*.

After a precise microbial diagnosis, the coinfected lesions were successfully treated with antibacterial (rifampicin/trimethoprim) and antiprotozoal agents (meglumine antimoniate). We think that antiprotozoal treatment was essential for skin healing, but a concomitant antibacterial therapy helped to heal the ulcerative lesions.

In view of the pathogenic potential of *S*. *pneumoniae* and a high carriage rate in children, we expect to find more cases in skin infections; however, this kind of coinfection has never been reported before. We propose that *S*. *pneumoniae* infections are being underestimated in skin lesions of patients from *Leishmania* endemic areas, probably by a misidentification with other gram-positive cocci that may coinfect these ulcers (see Table 1).

In this paper, our purpose is to alert physicians and microbiologists to the diagnosis of bacterial coinfection in *Leishmania* lesions. We suggest the implementation of an accurate microbial diagnosis of skin infections to allow the detection of *S*. *pneumoniae* and other ulcer-infecting pathogens, which may cause complications in the antimicrobial treatment.

**Key Learning Points**

- Cutaneous leishmaniasis is the most common clinical disease caused by all the *Leishmania* species that are pathogenic to humans.
- Other bacterial coinfections of *Leishmania* lesions have been described, but this is the first report of coinfection with *Leishmania* and *S*. *pneumoniae*.
- *S*. *pneumoniae* is also able to cause skin infections. For that reason, the pneumococcal diagnosis could be underestimated in *Leishmania* lesions coinfected with bacterial pathogens, particularly in endemic areas.
- An accurate microbiological diagnosis of *Leishmania* coinfections is essential for a correct antimicrobial treatment of skin infections.
- Pentavalent antimonials, such as meglumine antimoniate, are considered the first-line antimicrobial therapy for the treatment of leishmaniasis.
